# Supplementary figures and images for: Dynamics and triggers of misinformation on vaccines
Source: PLoS One. 2025 Jan 15;20(1):e0316258. doi: 10.1371/journal.pone.0316258 (PMC11734983; doi:10.1371/journal.pone.0316258)

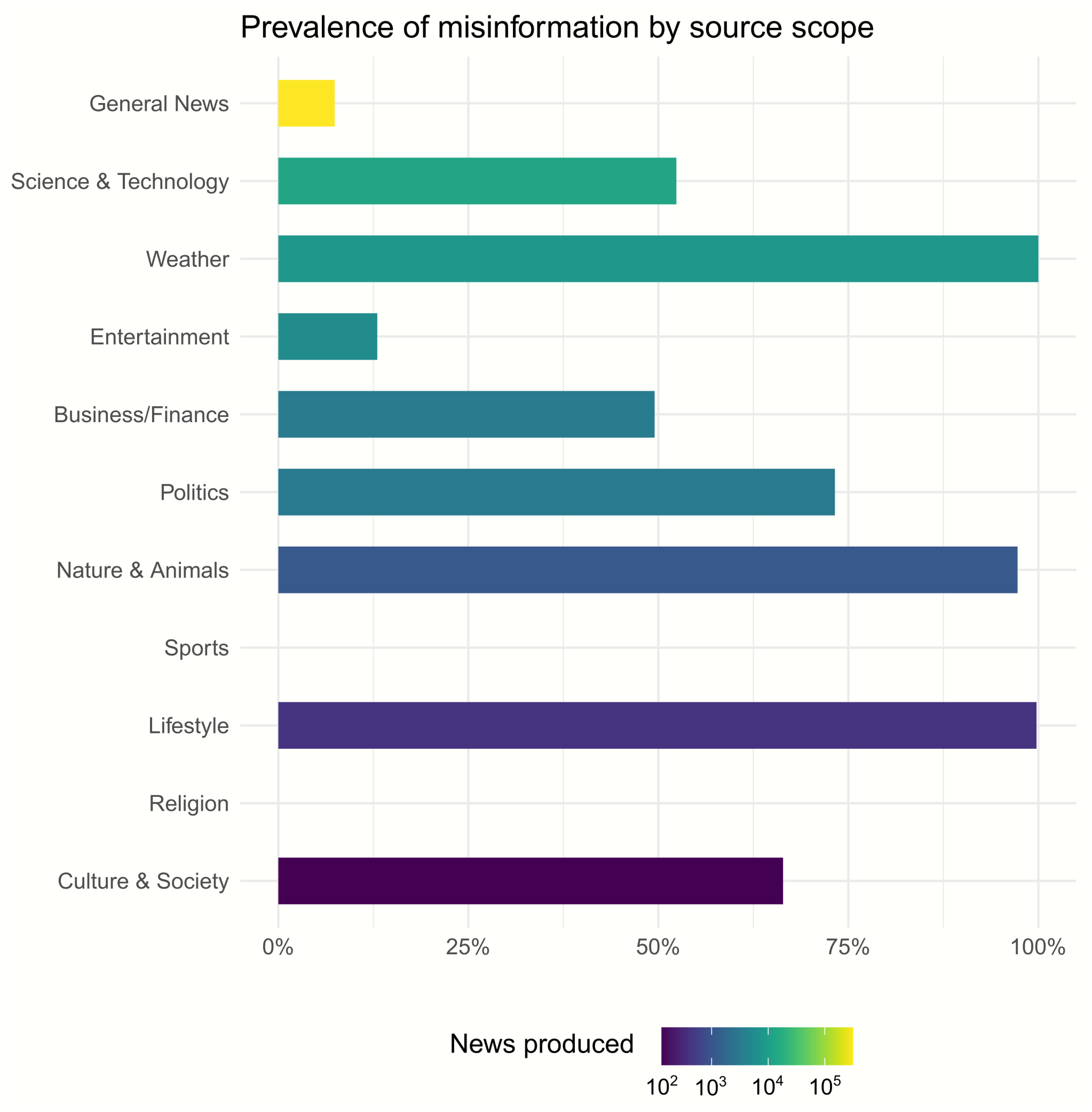

Supplement: S1 Fig — By exploiting the category classification provided by CrowdTangle and inspecting the page descriptions on Facebook, we extract the primary focus of all the 682 sources selected. Similar categories or those belonging to the same thematic area have been grouped together to streamline and condense the category list (e.g. pages dedicated to the topics of soccer and basketball, respectively, have merged into the Sports category). The final range of categories includes General News, Business/Finance, Culture & Society, Entertainment, Lifestyle, Nature & Animals, Politics, Religion, Science & Technology, Sports, Weather. Table shows the prevalence of misinformation on vaccines in relation to these categories. The color of the bars is associated with the total number of contents present in our dataset, regardless of the reliability of the source that produced them. Although General News is the primary focus for the questionable sources that produced most of the content on vaccines, these sources only contribute to one-tenth of the total vaccine-related content compared to reliable sources. Among specialized topics, while Entertainment, Sports, and Religion are rarely the main focus of vaccine misinformation, pages dedicated to Lifestyle, Nature & Animals, and Weather exclusively disseminate misinformation about vaccines. A clear prevalence of questionable sources is also evident in the Politics and Culture & Society categories, while a more balanced distribution is observed in the Business/Finance and Science & Technology categories. (TIF) [file pone.0316258.s001.tif]

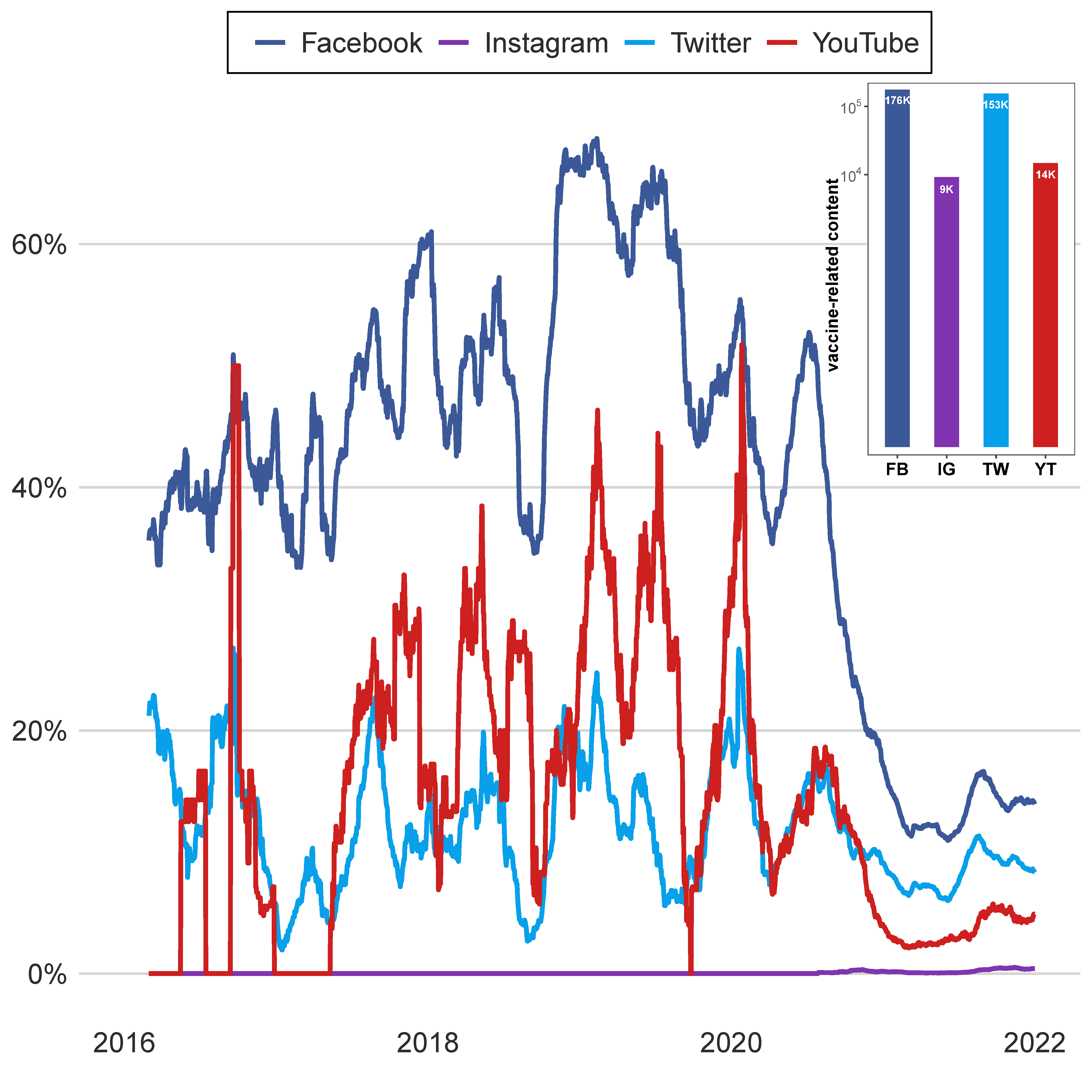

Supplement: S2 Fig — Plot shows the daily time-series, depicting the proportion of vaccine-related content originating from questionable sources in relation to the total volume of vaccine-related content (both from questionable and reliable sources). To bring out trends more clearly, for each social media, the time-series displayed concerns a 30-days simple moving average. Facebook stands out as the social media platform where vaccine misinformation is most prevalent. In the pre-pandemic period, approximately one out of every two vaccine-related contents published on the platform originates from questionable sources. In the subsequent period, the increased attention on the topic from reliable sources has brought the proportion back to more sustainable levels. Inset shows the quantity of vaccine-related content generated by the selected sources on each of the four social media platforms. Instagram and YouTube exhibit values that are one order of magnitude lower than those on Facebook and Twitter. (TIF) [file pone.0316258.s002.tif]

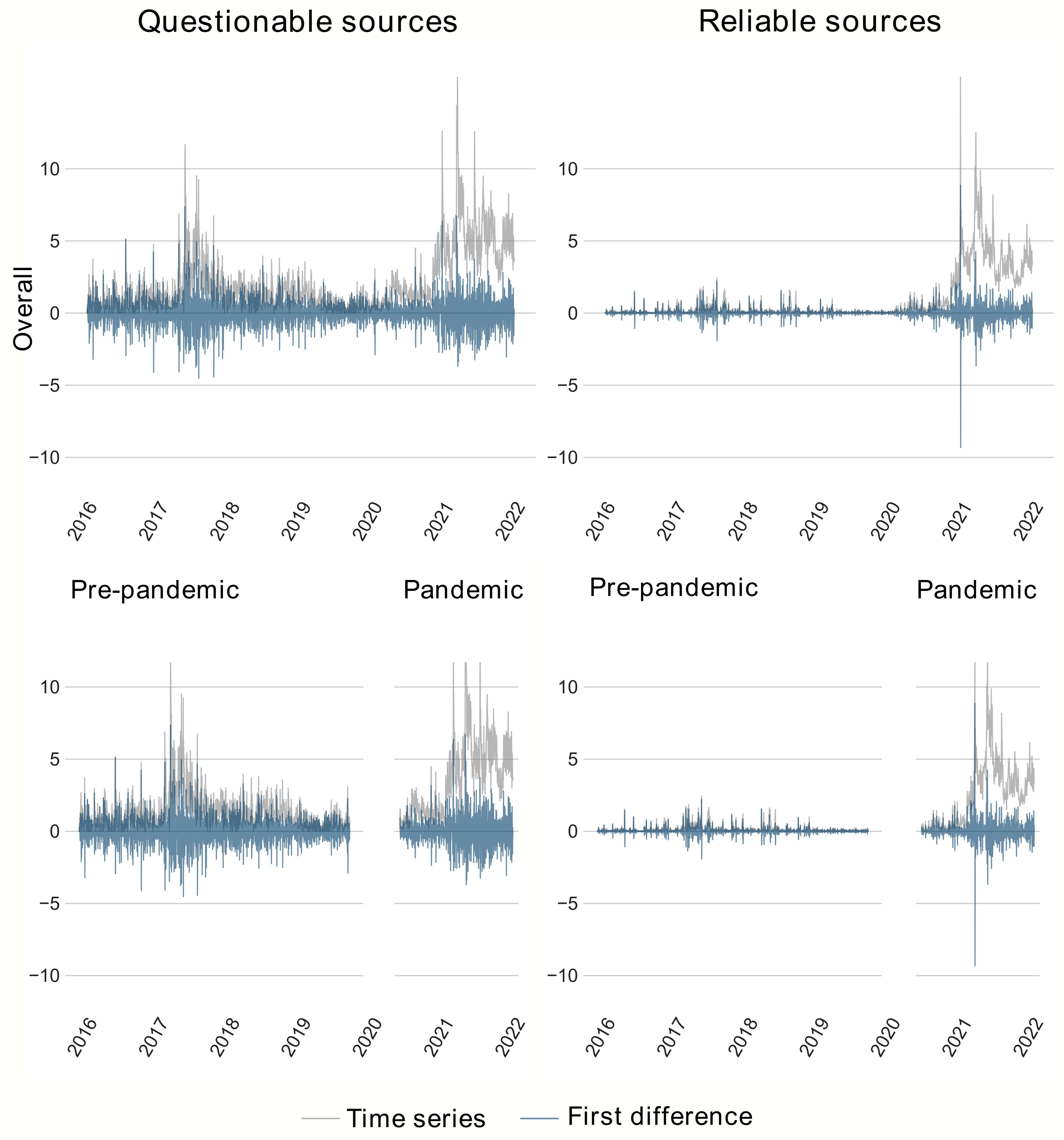

Supplement: S3 Fig — Graphics are broken down by period: Overall (1 January 2016–31 December 2021), pre-pandemic (1 January 2016–29 January 2020) and pandemic (30 January 2020–31 December 2021). (TIF) [file pone.0316258.s003.tif]

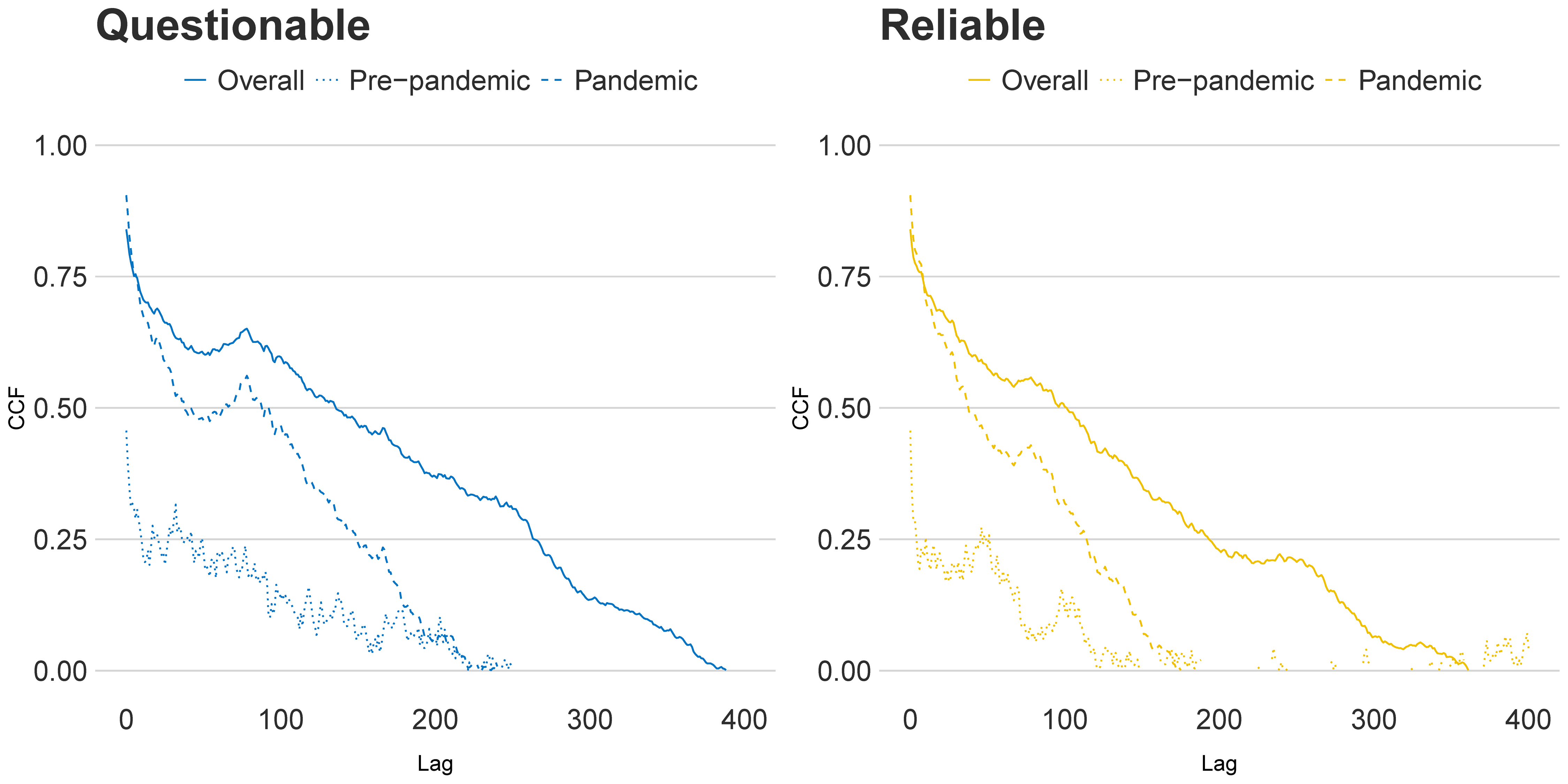

Supplement: S4 Fig — Cross-correlation function (CCF), i.e., ratio of covariance to root-mean variance, for daily time-series of the percentage of vaccine-related content from questionable (left panel) and reliable (right panel) sources, respectively. Plots concern the overall period (1 January 2016–31 December 2021) and the pre-pandemic (1 January 2016–29 January 2020) and pandemic (30 January 2020–31 December 2021) sub-periods, respectively. In the left (right) panel the lagged values refer to questionable (reliable) time-series. (TIF) [file pone.0316258.s004.tif]

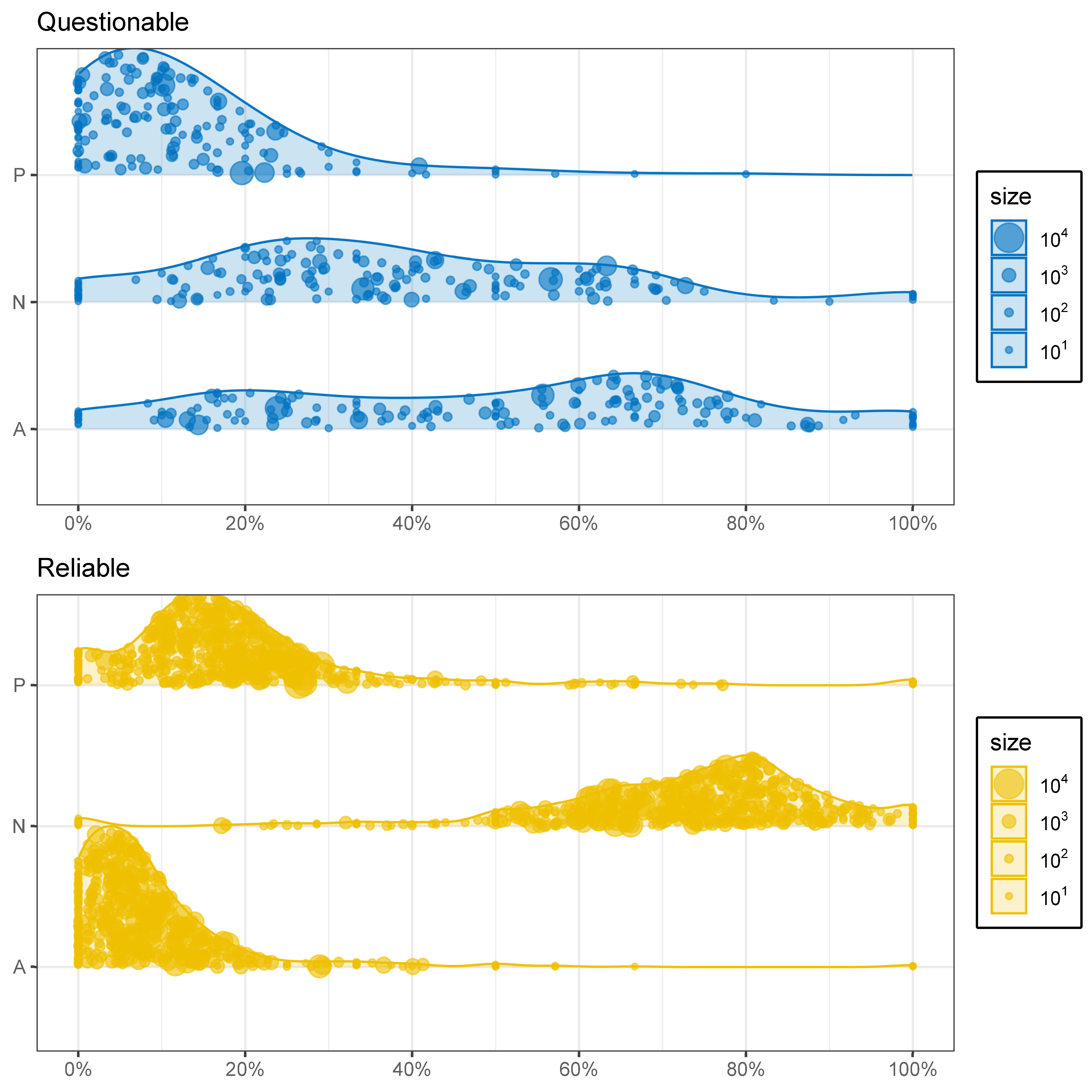

Supplement: S5 Fig — The observations underlying each empirical Probability Density Function curve represent the single sources and their sizes the corresponding amount of vaccine coverage. (TIF) [file pone.0316258.s005.tif]

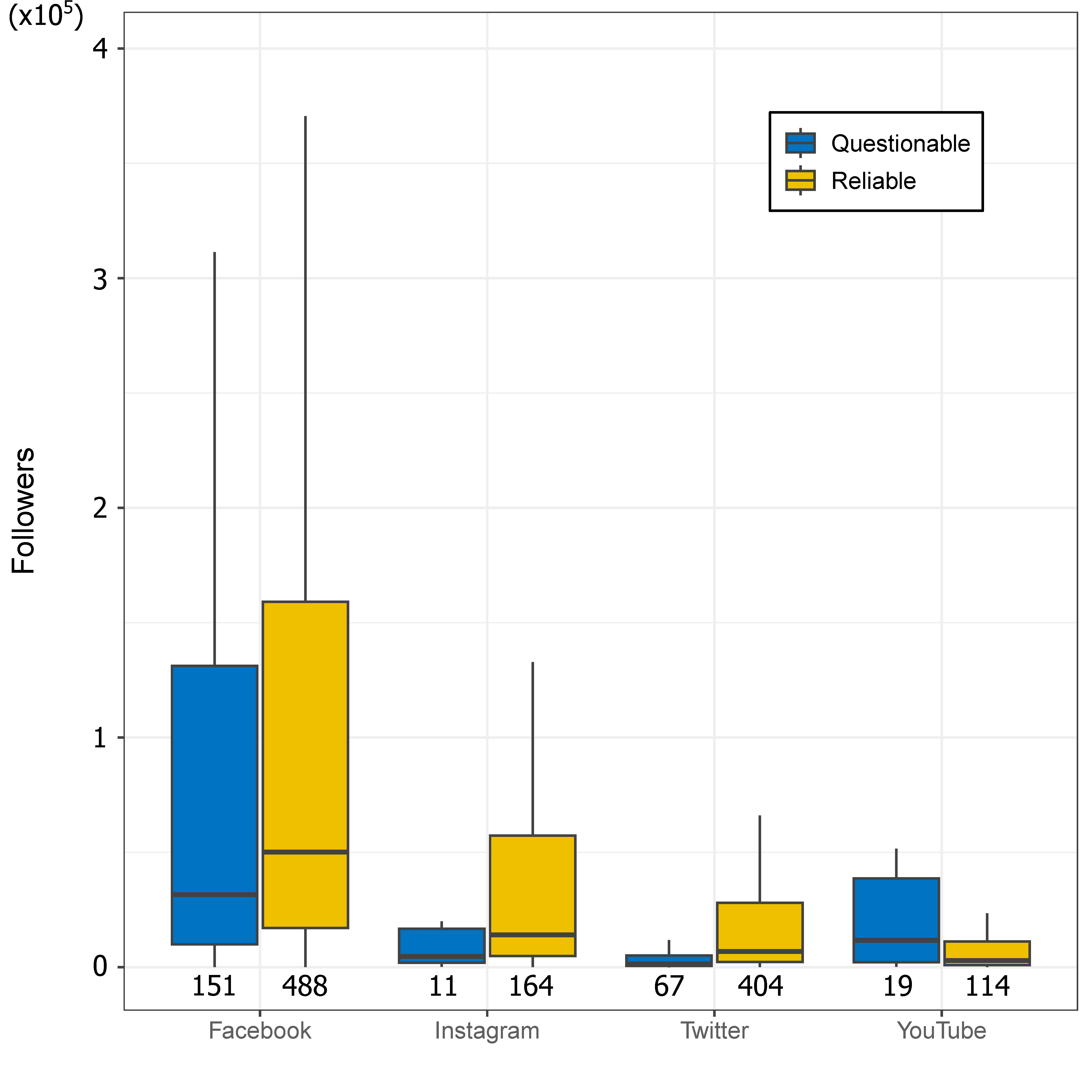

Supplement: S6 Fig — Outliers have been removed, and the y-axis range is limited to the 5th and 95th percentiles to highlight the central tendency of follower distribution within each platform. Median follower counts: Facebook 31,550 (Q), 50,088 (R); Instagram 4,736 (Q), 14,080 (R); Twitter 1,461 (Q), 6,883 (R); YouTube 11,736 (Q), 2,903 (R). The number of observations (i.e., sources) is indicated at the base of each boxplot. (TIF) [file pone.0316258.s006.tif]
