# Supplementary material for: Dynamics and triggers of misinformation on vaccines
Source: PLoS One. 2025 Jan 15;20(1):e0316258. doi: 10.1371/journal.pone.0316258 (PMC11734983; doi:10.1371/journal.pone.0316258)
Supplement: S1 Table — Table reports keywords divided by vaccine type: General, Covid-19 vaccines, Mandatory and Recommended vaccines (Law 119/2017), Other authorized and marketed vaccines. The special character * stands for zero or more non-whitespace characters. The list does not include the term ‘immunizzazione’ (immunization) and its variations, since anti-vaccination contents almost never talks about immunization [65,66]. Obviously, this is expected since anti-vaccination groups tend not to believe that vaccines confer immunity. Possible uses of the searched keywords in contexts other than the discussion on vaccines are, of course, quite rare. However, in the manual annotation process concerning stance and topic, we observed that the only critical cases involved content containing references to ‘latte vaccino’ (vaccine milk). Such content has been removed from the dataset. (DOCX) [file pone.0316258.s007.docx]

| Vaccine type | Searched keywords |
| --- | --- |
| General | vaccin${}^{*}$ OR vax OR no-vax |
| Covid-19 | pfizer OR pfizer/biontech OR mrnabnt162b2 OR bnt162b2 OR mrna-pfizer OR biontech OR bnt-162b2 OR mrna-bnt162b2 |
|  | astrazeneca OR vaxzevria OR vaxzevria/covid-19 OR chadox1 OR chadox1-s OR azd1222 OR (chadox1 ncov-2019) |
|  | moderna AND (covid OR coronavirus) OR spikevax OR mrna-1273 |
|  | janssen OR (johnson & johnson) OR johnson&johnson OR jnj-78436735 OR ad26.cov2.s OR j&j OR (j and j) OR (johnson and johnson) |
|  | sputnik OR gam-covid-vac OR gamaleya |
|  | reithera OR grad-cov2 |
| Mandatory (Law 119/2017) | (imovax polio) OR (imovax tetano) OR (engerix b) OR hbvaxpro OR varilrix OR varivax OR acthib OR hiberix OR diftetall OR revaxis OR boostrix OR triaxis OR tribaccine OR m-m-rvaxpro OR priorix OR polioboostrix OR polioinfanrix OR tetravac OR (triaxis polio) OR (priorix tetra) OR proquad OR hexyon OR (infanrix hexa) OR vaxelis |
| Recommended (Law 119/2017) | bexsero OR trumenba OR menjugate OR neisvac-c OR pneumovax OR (prevenar 13) OR synflorix OR rotateq OR rotarix OR |
| Other authorized and marketed | dukoral OR vaxchora OR ervebo OR dengvaxia OR (typhim vi) OR vivotif OR ticovac OR ixiaro OR (agrippal s1) OR fluad OR (influpozzi subunità) OR (influvac s) OR efluelda OR (fluad tetra) OR (fluarix tetra) OR (flucelvax tetra) OR (fluenz tetra) OR (influvac s tetra) OR (vaxigrip tetra) OR avaxim OR havrix OR vaqta OR (twinrix adulti) OR (twinrix pediatrico) OR fendrix OR rabipur OR shingrix OR zostavax OR stamaril OR menveo OR nimenrix OR cervarix OR (gardasil 9) |
