# Supplementary material for: Dynamics and triggers of misinformation on vaccines
Source: PLoS One. 2025 Jan 15;20(1):e0316258. doi: 10.1371/journal.pone.0316258 (PMC11734983; doi:10.1371/journal.pone.0316258)
Supplement: S2 Table — The measures are reported for the overall sample (1 January 2016–31 December 2021), together with the pre-pandemic (1 January 2016–29 January 2020) and pandemic (30 January 2020–31 December 2021) sub-periods. (DOCX) [file pone.0316258.s008.docx]

| Sourceset | Period | Mean | | St.Dev. | | Min | | Max | |
| --- | --- | --- | --- | --- | --- | --- | --- | --- | --- |
|  |  | TS | FD | TS | FD | TS | FD | TS | FD |
| Questionable | Overall | 2.051 | 0.002 | 2.074 | 1.061 | 0.000 | -4.525 | 16.346 | 7.366 |
|  | Pre-pandemic | 1.308 | 0.001 | 1.230 | 1.023 | 0.000 | -4.525 | 11.679 | 7.366 |
|  | Pandemic | 3.627 | 0.005 | 2.563 | 1.138 | 0.134 | -3.700 | 16.346 | 6.719 |
| Reliable | Overall | 0.921 | -0.009 | 1.700 | 0.499 | 0.000 | -9.316 | 16.344 | 8.871 |
|  | Pre-pandemic | 0.159 | 0.000 | 0.235 | 0.224 | 0.000 | -1.925 | 2.458 | 2.218 |
|  | Pandemic | 2.536 | 0.005 | 2.251 | 0.821 | 0.017 | -9.316 | 16.344 | 8.871 |
