# Supplementary material for: Dynamics and triggers of misinformation on vaccines
Source: PLoS One. 2025 Jan 15;20(1):e0316258. doi: 10.1371/journal.pone.0316258 (PMC11734983; doi:10.1371/journal.pone.0316258)
Supplement: S3 Table — The table reports the t-statistic of the ADF test statistics both in levels and on the first difference (FD). The ADF test is based on regressions with intercept. The null hypothesis for the test is non-stationarity. The estimates are reported for the overall period (1 January 2016–31 December 2021), together with the pre-pandemic (1 January 2016–29 January 2020) and pandemic (30 January 2020–31 December 2021) sub-periods. (DOCX) [file pone.0316258.s009.docx]

| Sourceset | Period | Levels | FD |
| --- | --- | --- | --- |
| Questionable | Overall | -8.802^***^ | -14.641^***^ |
|  | Pre-pandemic | -11.784^***^ | -12.140^***^ |
|  | Pandemic | -4.991^***^ | -4.587^***^ |
| Reliable | Overall | -5.634^***^ | -8.475^***^ |
|  | Pre-pandemic | -16.735^***^ | -3.436^***^ |
|  | Pandemic | -4.186^***^ | -4.072^***^ |
| ^***^*p*<0.001; ^**^*p*<0.01; ^*^*p*<0.05 | | | |
