# Supplementary material for: Dynamics and triggers of misinformation on vaccines
Source: PLoS One. 2025 Jan 15;20(1):e0316258. doi: 10.1371/journal.pone.0316258 (PMC11734983; doi:10.1371/journal.pone.0316258)
Supplement: S4 Table — Bins refer to the symbolic encoding performed to calculate the transfer entropy. The measures reported refer to both the overall sample (1 January 2016–31 December 2021), and the pre-pandemic (1 January 2016–29 January 2020) and pandemic (30 January 2020–31 December 2021) sub-periods. (DOCX) [file pone.0316258.s010.docx]

| Sourceset | Period | Bins | | | | |
| --- | --- | --- | --- | --- | --- | --- |
|  |  | (0,1] | (1,2] | (2,4] | (4,8] | (8,100] |
| Questionable | Overall | $q_{38}$ | $q_{67}$ | $q_{83}$ | $q_{98}$ | $q_{100}$ |
|  | Pre-pandemic | $q_{49}$ | $q_{81}$ | $q_{96}$ | $q_{99}$ | $q_{100}$ |
|  | Pandemic | $q_{14}$ | $q_{37}$ | $q_{57}$ | $q_{94}$ | $q_{100}$ |
| Reliable | Overall | $q_{79}$ | $q_{82}$ | $q_{92}$ | $q_{99}$ | $q_{100}$ |
|  | Pre-pandemic | $q_{98}$ | $q_{99}$ | $q_{100}$ | — | — |
|  | Pandemic | $q_{38}$ | $q_{44}$ | $q_{76}$ | $q_{97}$ | $q_{100}$ |
