# Supplementary material for: Dynamics and triggers of misinformation on vaccines
Source: PLoS One. 2025 Jan 15;20(1):e0316258. doi: 10.1371/journal.pone.0316258 (PMC11734983; doi:10.1371/journal.pone.0316258)
Supplement: S6 Table — Denoted with X the vaccine subject, Table shows the results of Mann-Whitney U test applied to the distributions of the out-engage factor P(Q,R;X;d) for the days d when it is in favor of source set Q and source set R, respectively. Since the distributions have values of opposite sign (See Eq (4) in the main text), the test is applied to the distributions of absolute values. Distributions are compared according to the period analyzed: Overall (1 January 2016–31 December 2021), pre-pandemic (1 January 2016–29 January 2020) and pandemic (30 January 2020–31 December 2021). (DOCX) [file pone.0316258.s012.docx]

| Period | Overperforming | Median | Mann-Whitney U test | |
| --- | --- | --- | --- | --- |
|  | sourceset |  | Statistic | $p$-value |
| Overall | Questionable | 14.21 | 41495 | $o({10}^{-45})$^***^ |
|  | Reliable | -2.23 |  |  |
| Pre-pandemic | Questionable | 26.20 | 21907 | $o({10}^{-42})$^***^ |
|  | Reliable | -2.28 |  |  |
| Pandemic | Questionable | 8.23 | 787 | $o({10}^{-08})$^***^ |
|  | Reliable | -1.30 |  |  |
| ^***^*p<0.001*; ^**^*p<0.01*; ^*^*p<0.05* | | | | |
