# Supplementary material for: Dynamics and triggers of misinformation on vaccines
Source: PLoS One. 2025 Jan 15;20(1):e0316258. doi: 10.1371/journal.pone.0316258 (PMC11734983; doi:10.1371/journal.pone.0316258)
Supplement: S9 Table — Reported data are estimates for intercept (α) and slope (β), corresponding confidence interval (95%) and significance, number of observations (months in the analyzed period), R2/R2adj. (DOCX) [file pone.0316258.s015.docx]

| Topic | $\alpha$ | | | $\beta$ | | | Obs. | $R^{2}/R^{2}adj$ |
| --- | --- | --- | --- | --- | --- | --- | --- | --- |
|  | Estimates | C. I. (95%) | $p$-value | Estimates | C. I. (95%) | $p$-value |  |  |
| Adm | -0.14 | [-0.21, -0.07] | $1.3e-04$*** | 3.35 | [2.61, 4.09] | $2.5e-13$*** | 72 | 0.54/0.53 |
| Bus | 0.12 | [-0.14, 0.37] | $3.7e-01$*** | 2.38 | [1.87, 2.88] | $5.4e-14$*** | 72 | 0.56/0.55 |
| Eff | 0.41 | [ 0.20, 0.63] | $2.7e-04$*** | 5.00 | [4.06, 5.94] | $3.3e-16$*** | 72 | 0.62/0.61 |
| Leg | 0.26 | [-0.01, 0.54] | $5.7e-02$*** | 2.99 | [2.11, 3.87] | $2.9e-09$*** | 72 | 0.40/0.39 |
| Saf | 0.64 | [-0.07, 1.35] | $7.8e-02$*** | 3.69 | [2.13, 5.25] | $1.2e-05$*** | 72 | 0.24/0.23 |
| Oth | -0.03 | [-0.12, 0.06] | $4.6e-01$*** | 2.69 | [1.89, 3.48] | $4.0e-09$*** | 72 | 0.39/0.38 |
| ^***^*p<0.001*; ^**^*p<0.01*; ^*^*p<0.05* | | | | | | | | |
