# Supplementary material for: Dynamics and triggers of misinformation on vaccines
Source: PLoS One. 2025 Jan 15;20(1):e0316258. doi: 10.1371/journal.pone.0316258 (PMC11734983; doi:10.1371/journal.pone.0316258)
Supplement: S10 Table — Data are divided by source category (questionable and reliable) and period analyzed (Overall 1 January 2016–31 December 2021, Pre-pandemic 1 January 2016–29 January 2020, Pandemic 30 January 2020–31 December 2021). Percentages are further divided according to the stance conveyed by the corresponding content (A = anti-vax, N = neutral, P = pro-vax). (DOCX) [file pone.0316258.s016.docx]

|  | Topic | Overall (%) | | | | Pre-pandemic (%) | | | | Pandemic (%) | | | |
| --- | --- | --- | --- | --- | --- | --- | --- | --- | --- | --- | --- | --- | --- |
|  |  | A | N | P | Σ | A | N | P | Σ | A | N | P | Σ |
| Questionable | Adm | 3.5 | 17.3 | 2.4 | 23.2 | 3.1 | 6.0 | 0.7 | 9.8 | 3.6 | 19.5 | 2.7 | 25.8 |
|  | Bus | 4.7 | 1.9 | 0.4 | 7.0 | 8.0 | 0.8 | 0.1 | 8.9 | 4.0 | 2.1 | 0.5 | 6.6 |
|  | Eff | 8.9 | 8.7 | 6.6 | 24.2 | 7.4 | 3.2 | 2.8 | 13.4 | 9.2 | 9.8 | 7.3 | 26.3 |
|  | Leg | 4.1 | 5.0 | 0.7 | 9.8 | 8.2 | 7.4 | 0.4 | 16.0 | 3.3 | 4.5 | 0.8 | 8.6 |
|  | Saf | 21.6 | 6.1 | 2.1 | 29.8 | 36.5 | 6.2 | 1.8 | 44.5 | 18.6 | 6.1 | 2.2 | 26.9 |
|  | Oth | 1.3 | 3.7 | 1.0 | 6.0 | 2.3 | 4.8 | 0.3 | 7.4 | 1.1 | 3.5 | 1.2 | 5.8 |
|  | Σ | 44.1 | 42.7 | 13.2 | 100.0 | 65.5 | 28.4 | 6.1 | 100.0 | 39.8 | 45.5 | 14.7 | 100.0 |
| Reliable | Adm | 1.9 | 45.7 | 6.1 | 53.7 | 1.0 | 20.0 | 3.5 | 24.5 | 2.0 | 47.1 | 6.2 | 55.3 |
|  | Bus | 0.9 | 2.1 | 0.6 | 3.6 | 0.6 | 1.3 | 0.7 | 2.6 | 0.9 | 2.2 | 0.6 | 3.7 |
|  | Eff | 2.3 | 7.8 | 8.8 | 18.9 | 1.1 | 6.4 | 12.0 | 19.5 | 2.4 | 7.9 | 8.6 | 18.9 |
|  | Leg | 0.7 | 5.4 | 0.8 | 6.9 | 1.5 | 24.0 | 2.0 | 27.5 | 0.7 | 4.4 | 0.6 | 5.7 |
|  | Saf | 3.8 | 5.4 | 2.8 | 12.0 | 3.1 | 5.7 | 6.1 | 14.9 | 3.8 | 5.4 | 2.6 | 11.8 |
|  | Oth | 0.3 | 3.6 | 1.0 | 4.9 | 0.5 | 8.8 | 1.7 | 11.0 | 0.3 | 3.3 | 1.0 | 4.6 |
|  | Σ | 9.9 | 70.1 | 20.0 | 100.0 | 7.8 | 66.2 | 26.0 | 100.0 | 10.1 | 70.3 | 19.6 | 100.0 |
