# Supplementary material for: Dynamics and triggers of misinformation on vaccines
Source: PLoS One. 2025 Jan 15;20(1):e0316258. doi: 10.1371/journal.pone.0316258 (PMC11734983; doi:10.1371/journal.pone.0316258)
Supplement: S11 Table — Confusion matrices for the evaluation set with respect to stance: Between the annotators and the model (a), and between annotators (b). The performance measures, Acc and F1, are calculated from these matrices. The axes show the possible labels (A = anti-vax, N = neutral, P = pro-vax). (DOCX) [file pone.0316258.s017.docx]

| **(a)** Stance model.   \| Label \| A \| N \| P \| Σ \| \| --- \| --- \| --- \| --- \| --- \| \| A \| 2601 \| 253 \| 66 \| 2920 \| \| N \| 292 \| 2942 \| 317 \| 3551 \| \| P \| 70 \| 122 \| 2636 \| 2828 \| \| Σ \| 2963 \| 3317 \| 3019 \| 9299 \| | **(b)** Annotators.   \| Label \| A \| N \| P \| Σ \| \| --- \| --- \| --- \| --- \| --- \| \| A \| 279 \| 22 \| 8 \| 309 \| \| N \| 24 \| 302 \| 27 \| 353 \| \| P \| 8 \| 19 \| 260 \| 287 \| \| Σ \| 311 \| 343 \| 295 \| 949 \| |
| --- | --- | --- | --- | --- | --- | --- | --- | --- | --- | --- | --- | --- | --- | --- | --- | --- | --- | --- | --- | --- | --- | --- | --- | --- | --- | --- | --- | --- | --- | --- | --- | --- | --- | --- | --- | --- | --- | --- | --- | --- | --- | --- | --- | --- | --- | --- | --- | --- | --- | --- | --- |
