# Supplementary material for: Dynamics and triggers of misinformation on vaccines
Source: PLoS One. 2025 Jan 15;20(1):e0316258. doi: 10.1371/journal.pone.0316258 (PMC11734983; doi:10.1371/journal.pone.0316258)
Supplement: S12 Table — Confusion matrices for the evaluation set with respect to topic: Between the annotators and the model (a), and between annotators (b). The performance measures, Acc and F1, are calculated from these matrices. The axes show the possible labels (Adm = administration of vaccines, Bus = vaccine business, Eff = effectiveness of vaccination, Leg = legal issues, Saf = safety concerns, Oth = other). (DOCX) [file pone.0316258.s018.docx]

| **(a)** Topic model.   \| Label \| Adm \| Bus \| Eff \| Leg \| Saf \| Oth \| Σ \| \| --- \| --- \| --- \| --- \| --- \| --- \| --- \| --- \| \| Adm \| 2150 \| 21 \| 130 \| 43 \| 100 \| 32 \| 2476 \| \| Bus \| 23 \| 350 \| 18 \| 5 \| 11 \| 5 \| 412 \| \| Eff \| 86 \| 19 \| 2108 \| 9 \| 110 \| 11 \| 2343 \| \| Leg \| 53 \| 22 \| 17 \| 538 \| 37 \| 31 \| 698 \| \| Saf \| 57 \| 10 \| 84 \| 23 \| 2723 \| 26 \| 2923 \| \| Oth \| 40 \| 5 \| 18 \| 23 \| 43 \| 318 \| 447 \| \| Σ \| 2409 \| 427 \| 2375 \| 641 \| 3024 \| 423 \| 9299 \| |
| --- | --- | --- | --- | --- | --- | --- | --- | --- | --- | --- | --- | --- | --- | --- | --- | --- | --- | --- | --- | --- | --- | --- | --- | --- | --- | --- | --- | --- | --- | --- | --- | --- | --- | --- | --- | --- | --- | --- | --- | --- | --- | --- | --- | --- | --- | --- | --- | --- | --- | --- | --- | --- | --- | --- | --- | --- | --- | --- | --- | --- | --- | --- | --- | --- |
| **(b)** Annotators.   \| Label \| Adm \| Bus \| Eff \| Leg \| Saf \| Oth \| Σ \| \| --- \| --- \| --- \| --- \| --- \| --- \| --- \| --- \| \| Adm \| 213 \| 4 \| 14 \| 5 \| 12 \| 4 \| 252 \| \| Bus \| 3 \| 33 \| 2 \| 1 \| 2 \| 1 \| 42 \| \| Eff \| 9 \| 2 \| 211 \| 0 \| 12 \| 2 \| 236 \| \| Leg \| 8 \| 2 \| 1 \| 57 \| 5 \| 5 \| 78 \| \| Saf \| 4 \| 0 \| 8 \| 2 \| 279 \| 2 \| 295 \| \| Oth \| 4 \| 1 \| 2 \| 3 \| 4 \| 32 \| 46 \| \| Σ \| 241 \| 42 \| 238 \| 68 \| 314 \| 46 \| 949 \| |
