# Supplementary material for: Dynamics and triggers of misinformation on vaccines
Source: PLoS One. 2025 Jan 15;20(1):e0316258. doi: 10.1371/journal.pone.0316258 (PMC11734983; doi:10.1371/journal.pone.0316258)
Supplement: S13 Table — Results refer to the model yit=α+βxi+γzit+θi+ϵit,i=1,2,…,N,t=1,2,…,T, where i is the news source identifier, t is time (days since January 1st, 2016), yit is the user engagement on vaccine-related content from source i on day t,xi is a vector of time-independent source-related variables (e.g., the factualness classification: Reliable or questionable), zit are time-dependent factors (e.g., number, type and topic of news content), and θi,ϵit are error terms with auto-correlated component. Being the panel data unbalanced and T>N, we choose a model with autocorrelated disturbances. Robust estimators of variance were also estimated, and the results are confirmed [67,68]. Model I results show that content conveying pro-vax stance decreases user engagement (elasticity -0.03%) while anti-vax stance increases it (elasticity 0.5%), especially when it is proposed by questionable sources (Model II, coefficient of the interactive variable 1.34%). Conversely, when questionable sources convey pro-vax stance, engagement drops by 0.3%. If we focus on topics (Models III and IV), “safety concerns” is the one that elicits the greatest reactions (0.4%), especially when content comes from questionable sources (maximum elasticity 1.1%). Engagement drops instead when questionable sources cover the topic of effectiveness of vaccination. (DOCX) [file pone.0316258.s019.docx]

| **Model**  Variable | **Stance**  (I) | | **Stance**  (II) | | **Topic**  (III) | | **Topic**  (IV) | |
| --- | --- | --- | --- | --- | --- | --- | --- | --- |
| **Source’s factualness** | | | | | | | | |
| Questionable (D) | .000778^***^ | (.000245) | .000464^*^ | (.000241) | .000849^***^ | (000246) | .000567^**^ | (.000242) |
| **Content** | | | | | | | | |
| Anti-vax (ln) | .005410^***^ | (.000128) | .000349^**^ | (.000161) |  |  |  |  |
| Quest·Anti-vax (D·ln) |  |  | .013400^***^ | (.000262) |  |  |  |  |
| Pro-vax (ln) | -.000282^**^ | (.000111) | .000680^***^ | (.000117) |  |  |  |  |
| Quest·Pro-vax (D·ln) |  |  | -.002880^***^ | (.000383) |  |  |  |  |
| **Topic** | | | | | | | | |
| Administration (ln) |  |  |  |  | -000317^***^ | (.000007) | .000355^***^ | (.000008) |
| Quest·Admin (D·ln) |  |  |  |  |  |  | -.00363^***^ | (.000311) |
| Business (ln) |  |  |  |  | .000946^***^ | (.000227) | -.000008 | (.000256) |
| Quest·Business (D·ln) |  |  |  |  |  |  | .005170^***^ | (.000552) |
| Legal (ln) |  |  |  |  | .00222^***^ | (.000173) | .000292 | (.000189) |
| Quest·Legal (D·ln) |  |  |  |  |  |  | .0113^***^ | (.000461) |
| Effectiveness (ln) |  |  |  |  | .000000 | (.000115) | .000348^***^ | (.000125) |
| Quest·Effective (D·ln) |  |  |  |  |  |  | -.00133^***^ | (.000316) |
| Safety (ln) |  |  |  |  | .00357^***^ | (.000130) | .000249^*^ | (.000151) |
| Quest·Safety (D·ln) |  |  |  |  |  |  | .0125^***^ | (.000297) |
| Constant | -.000136 | (.000143) | -.000110 | (.000141) | -.000143 | (.000143) | -.000125 | (.000142) |
| **Controls** | | | | | | | | |
| Time(weekday,month,year) | YES | | YES | | YES | | YES | |
| **Summary stats** | | | | | | | | |
| Observations | 1,378,129 | | 1,378,129 | | 1,378,129 | | 1,378,129 | |
| Number of sources | 680 | | 680 | | 680 | | 680 | |
| $\chi^{2}$ (dof) | 1961.98^***^ | (26) | 4609.65^***^ | (28) | 1218.21^***^ | (29) | 4035.04^***^ | (34) |
| $R^{2}$ between | .1135 | | .2079 | | .0787 | | .2141 | |
| The baselines of the estimates are "neutral" for models on stance (I and II), and "other" (e.g., events, related news) for models on topics (III and IV). The dependent variable is the natural logarithm of the engagement. For each variable, the coefficient, the level of significance (^***^1%; ^**^5%; ^*^10%), and the standard error (in parentheses) are reported. | | | | | | | | |
