# Supplementary material for: Dynamics and triggers of misinformation on vaccines
Source: PLoS One. 2025 Jan 15;20(1):e0316258. doi: 10.1371/journal.pone.0316258 (PMC11734983; doi:10.1371/journal.pone.0316258)
Supplement: Attachment — Submitted filename: Response to Reviewers.docx [file pone.0316258.s020.docx]

PONE-D-22-33837

**Dynamics and triggers of misinformation on vaccines**

(Previously titled “Dynamics of (mis)information flow and engaging power of narratives”)

**Response to Reviewers**

**Reviewer #1**: In short, the authors need to dig in a lot more to the methods to better understand the biases introduced in their data collection on both production and consumption. Conditional on that, I find the key findings in Figure 3 and 4 compelling, but really need the authors to update their figure before I can really confirm.

*We are grateful to the reviewer for recognizing the value of our work and offering insightful comments that have greatly enhanced the quality of our work. Following are our responses to these comments, along with references to the main text.*

I understand the style of moving methods into a follow-up section after the discussion, but the paper is unreadable due to too much key parts in the methods section.

*We thank the reviewer for suggesting us a more suitable arrangement of the various sections to enhance the readability of the paper. Indeed, the manuscript was quite unreadable following the original order. “Materials and methods” section has been moved before “Results and discussion”.*

1) I am still very confused about the core data collection methods, even after re-reading the introduction and methods several times. What I think the authors did was go onto Crowtangle and the API for Twitter and YouTube and search for keywords around vaccines. They then took content that was from publications (discarding any posts) and graded quality or not based on publisher level. But, Figure 1 shows percent of all content? So, maybe they collect all links?

*We sincerely thank the reviewer for bringing up this point. We had indeed explained the data collection methods very poorly. What we did is first defining a comprehensive set of sources that was representative of the Italian news ecosystem. Then, we gathered all the content on vaccines published by these sources on Facebook, Instagram, Twitter and YouTube between 2016 and 2021. To do this, we searched for content whose textual parts (message, image text, or any other description) matched an exhaustive list of vaccine-related keywords. Data from Facebook and Instagram were collected through CrowdTangle. Data from Twitter and YouTube were gathered by means of their official APIs. Twitter API was accessed through an academic account before the limitations introduced by the new management. These APIs also enable us to obtain time series metrics for single sources or sets of sources. Hence, to get an accurate estimate of how much attention questionable sources and reliable sources have dedicated, respectively, to the topic of vaccines compared to the rest of the covered topics, we downloaded the time series of the total contents published and the total interactions gained by the two source sets. The “Data collection” section has been partially revised to clarify the method used to build our dataset. Additionally, a significant portion of the Introduction (lines 80-88 on page 4) has been dedicated to this subject.*

Either way, way: what are they doing to validate their keywords (recall and precision of their labelling). Presumably they get some idea of false positives when hand coding for topic and position.

*The complete list of keywords used for filtering content related to vaccines consists of few general terms (e.g. vaccine, vaccination), together with vaccine names/brands the various types of vaccine and the corresponding names of the drugs available in Italy, as retrieved from the website of the Italian Medicines Agency. We assumed that each content whose textual part matched at least one of these keywords was related to the vaccine subject. Possible uses of these terms in contexts other than the discussion on vaccines are, of course, quite rare. However, in the manual annotation process, we observed that the only critical cases involved content containing references to 'latte vaccino' (vaccine milk). Such content has been removed from the dataset. This specification was added to the description of S2 Table.*

And, what are they doing to deal with issues of images and videos.

*The post types in our dataset, as conveyed by the four social media platforms through the aforementioned APIs, include:*

| *carousel* | *event* | *igtv* | *image* | *link* | *music* | *note* | *photo* | *status* | *video* |
| --- | --- | --- | --- | --- | --- | --- | --- | --- | --- |
| *812* | *1* | *713* | *7287* | *238683* | *2* | *5* | *69770* | *8244* | *27950* |

*We specifically examined the occurrence of selected keywords solely within the textual data associated with these posts. Therefore, images or videos are also addressed through elements such as image text or any other description.*

2) The authors are using Facebook, Instagram, Twitter, and YouTube to measure engagement with content: what exactly are they measuring? In the methods they recognize that these platforms are measuring very different types of engagement, and they are measuring them differently.

*The news items collected were processed regardless of the social media where they were published. In other words, the contents* $c\left( s;T \right)$ *published by a news source* $s$ *during the time span* $T$ *refer to the totality of its Facebook posts, Instagram media, Twitter tweets and YouTube videos. Analogously, the user interactions* $i\left( s;T \right)$ *include the totality of actions taken on* $c\left( s;T \right)$ *throughout* $T$*: comments, shares, likes and other reactions (angry, haha, love, sad, wow) on Facebook posts; comments and likes on Instagram media; replies, retweets and likes on Twitter tweets; comments, likes and dislikes on YouTube videos.*

*In other words, as a proxy for the reach of a particular type of content (in our case, reliable or questionable), we considered all possible interactions users had with that content on each of the four social media platforms, evaluating overall engagement.*

*Further, we added the S2 Fig. providing a comparison of misinformation prevalence across the four social media platforms (See also lines 351-355 at page 15).*

For example, CrowdTangle only includes URL with greater than 100 public shares. What the author are capturing is very different from views and biased, in many ways, towards click-bait or more sensational, low quality information. The authors should address this!

*Social Dark Matter (SDM), referring to content that individuals have a strong incentive to conceal, poses a challenge in terms of quantification. When considering social media platforms, SDM can be thought of as views to content rather than the actual interactions it garners. Unfortunately, obtaining precise information about views on social networks, at least during the analyzed period, is not feasible. Looking beyond mere interest, it would be exceptionally impactful to conduct analyses comparing views and visible engagement on social media, particularly with the perspective of unbiased datasets that can explore the actual effects of misinformation on vaccine hesitancy. In our analysis, apart from quantitatively comparing the prevalence and spread of vaccine-related content from news sources of opposing quality (questionable and reliable), we also sought to investigate the roots of possible differences in terms of opinions conveyed and topics covered. Furthermore, we explored instances where one perspective outweighs the other in terms of user engagement. This is why we relied on total visible engagement as a proxy for the reach of each piece of content. In other words, we rely on visible interactions rather than views because we aim to measure whether an opinion is not only viewed but also shared.*

*Further, we did not find any evidence supporting the 100-public-share threshold for inclusion of Facebook URL in Crowdtangle. We are aware of this limitation in the posts present in the Facebook URLs dataset (*[*https://socialscience.one/blog/unprecedented-facebook-urls-dataset-now-available-research-through-social-science-one*](https://socialscience.one/blog/unprecedented-facebook-urls-dataset-now-available-research-through-social-science-one)*), but it does not seem to apply to CrowdTangle data [A], as also indicated by the following figure depicting the Complementary Cumulative Distribution Function (CCDF) of the number of shares per Facebook (FB) post in the dataset gathered from CrowdTangle. Approximately one-tenth of these contents received more than 100 shares.*

*[A] Allen, J., Mobius, M., Rothschild D. M., & Watts, D. J. (2021). Research note: Examining potential bias in large-scale censored data. Harvard Kennedy School (HKS) Misinformation Review.* [*https://doi.org/10.37016/mr-2020-74*](https://doi.org/10.37016/mr-2020-74)

3) In Figure 1 (and assorted tables): percent of all content is highly related to how they are defining the corpus. This is especially important for reliable content: are the authors including sports and entertainment sections of the papers? Questionable content is more likely constrained to politically oriented publications?

*By exploiting the category classification provided by CrowdTangle and inspecting the page descriptions on Facebook, we extract the primary focus of all the 682 sources selected. S1 Fig. shows the prevalence of misinformation on vaccines in relation to these categories.
Among specialized topics, Entertainment, Sports, and Religion are rarely the main focus of questionable sources. Conversely, pages dedicated to Lifestyle, Nature & Animals, and Weather that have published content about vaccines during the period analysed are all categorized within the questionable source set. A clear prevalence of questionable sources is also evident in the Politics and Culture & Society categories, while a more balanced distribution is observed in the Business/Finance and Science & Technology categories.
While these categories may be distinctive for one of the two source sets, "General News" remains the primary focus for both questionable and reliable sources. Questionable sources within this category represent approximately 57% of the total questionable sources in our dataset and have produced content approximately two orders of magnitude greater than questionable sources belonging to the aforementioned categories. The in-text reference can be found at lines 158-159 of page 6.*

4) For Figure 2 the authors are trying to measure how engaging vaccine related content is within a publication compared to other content, and within vaccine related content by follower. I find the terms confusing, but that is obviously not critical. The discovery of content is conditional on engagement making these measures troubled. For an article to be discovered through CrowdTangle it had to be publicly shared 100+ times! For the “outside” perspective, the authors are using “followers”, presumably on the platform? How well does this corelate with consumption and engagement? I assume low quality publications get followed very differently than high-quality publications.

*We thank the reviewer for raising this point which has been addressed in the text by emphasizing the importance of considering normalization by followers in an engagement metric to control for potential scaling effects (see lines 587-590 of page 23).
The procedure to compute engagement with X-related content, where X is a set of features, and perform a comparison between questionable (Q) and reliable (R) source sets, is exhaustively described in the section Materials and methods. Basically, we define engagement with the X-related content from source set S during T (i.e., C(S;X;T)) as E(S;X;T)=I(S;X;T)/(C(S;X;T)⋅F(S;T)), where I(S;X;T) is the total interactions with that content and F(S;T) is the average number of followers of the social media accounts (FB, IG, TW, YT) of S which were active during T. Then, we explore both the inside and outside perspectives through the out-engage factor defined by Equation (4). In the former case, we calculate the ratio E(S;X;T)/E(S;X’;T) both for S=Q and S=R, where X stands for vaccine-related and X’ for any other subject. In the latter case, we calculate E(Q;X;T)/E(R;X;T). We also cite here the piece of text from lines 433-440 of page 18: “It is worth noting that while normalization by followers does not affect the out-engage factor formula from the inside perspective (it contributes twice equally but inversely), it has a huge impact on the same formula from the outside perspective, either by reducing the contribution of news content from sources with a large follower base, or by amplifying the contribution of news content from sources with a small follower base [59]. This approach prevents us from the risk of confusing scale effects with the real user engagement (i.e., mainstream news media have more audience than questionable sources and therefore trigger more user interactions, all things being equal).”*

5) The model is predicting the labeled position (1 of 3) with 88% in the evaluation set? That is sort of shockingly good. Conditional on all my previous concerns over the corpus, I find the left side of Figure 3 engaging and meaningful.

*We are particularly pleased with the accuracy of the models for classifying stance and topic, which is mainly due to the extensive manual annotation work. The resources are publicly available for further research on the subject at the osf repository* [*https://osf.io/nsmk3/*](https://osf.io/nsmk3/)*.*

*The right side of Figure 3 has been partially revised to make the comparison between engagement obtained from the two source sets, with respect to the different expressed stances, more straightforward.*

6) Similarly, accepting a sufficient level of accuracy, Figure 4 is also has the potential be meaningful. Interesting to see the different topics by publication type.

*Figure 3 shows that if anti-vax rhetoric is distinctive of questionable sources both in terms of content produced and engagement gained, such quantities are distinctive of reliable sources when expressing uplifting perspectives. By leveraging the outcomes derived from applying both the stance and topic models to the entire vaccine dataset, we investigate the relationship between the discrepancy in coverage between anti-vax content from questionable sources and pro-vax content from reliable sources, and the corresponding out-engage factor for each topic. This led to a complete revision of Figure 4, which now displays the log-linear relationship between the two variables for each topic.*

**Reviewer #2**: By identifying impact of misinformation authors are contributing a good quality of research but few things needs to be considered while representation of the paper.

*We sincerely appreciate the reviewer for acknowledging our work and for providing valuable comments that have significantly improved it. Below is a brief response to these comments with references to the main text.*

1. Highlighting the state of the art contribution is important.

*We appreciate the reviewer for bringing up this point. Indeed, the initial version of the manuscript did not adequately highlight the numerous contributions of our work to the existing literature on the subject. These contributions span various aspects, including methodological advancements such as:*

*- a comprehensive definition of engagement that controls for possible scaling effects (see section “User engagement and overperforming content”, lines 433-440, and lines 587-590);
- the provision of resources such as two models for classifying stance and topic in vaccine-related content in a language that receives less attention in scientific research compared to languages like English (see section “Modelling stance conveyed and topic discussed in vaccine-related content”, lines 29-31, lines 99-103, lines 479-484, and lines 614-618);*

*and significant findings such as:*

*- the identification of misinformation as an autonomous force capable of overwhelming the production of vaccine-related content from reliable media;
- the significantly higher engagement of questionable sources compared to reliable ones;
- the effectiveness of consistent and thorough counter-narratives to limit the spread of vaccine misinformation.*

2. Abstract is not summarizing the exact unique contribution and goal of the work is not clear from abstract as well as introduction.

*Abstract and introduction have been thoroughly revised. By addressing the points raised by the reviewers, the revised sections now provide a clearer overview of the study's objectives, methods, and findings.*

3. It requires strengthening paper presentation in several parts, including an introduction (Where is the novelty of the methods, explain it clearly)

*As stressed above, we have revised the introduction as well as the other sections to enhance the paper's readability and emphasize the contribution of the work to the existing literature on misinformation. See previous comments.*

4. The proposed model should include the proposed formulation

*Thank you for your comment which gives us the opportunity to clarify this aspect.
The model referred to in the paper is a deep-learning model for text classification. Details are included in section “Modelling stance conveyed and topic discussed in vaccine-related content”, and where necessary include references to the relevant papers. Other models are considered in the analysis:*

*- log-linear models describing the relationship between the discrepancy in coverage between anti-vax content from questionable sources and pro-vax content from reliable sources, and the corresponding out-engage factor for each topic (see Fig 4)
- econometric models describing the relationship between news source reliability, conveyed stance, discussed topic, and generated engagement (see S14 Fig.)*

*It's possible that the reviewer meant "method" instead of "model."
The method referred to in the paper is a comprehensive approach involving various facets, ranging from the collection of vaccine-related contents to their classification and analysis. These aspects are summarized in the introduction (lines 72-103) and further detailed in the dedicated section titled "Materials and methods."*

5. more emphasis is required on how the use of Deep learning model contributes to achieve results.

*We really appreciate the feedback. Indeed, the first submission of the manuscript did not adequately highlight the importance of leveraging deep learning models capable of classifying content based on specific features for which they are fine-tuned. In our case, the accuracy of stance and topic classifiers for vaccine-related content is particularly high, largely due to extensive manual annotation efforts. These aspects have been more thoroughly discussed and highlighted both in the dedicated section ("Modelling stance conveyed and topic discussed in vaccine-related content") and in the abstract, introduction, and "Fighting the spread of vaccine misinformation through compelling counter-narratives" section.*

6. The authors should add the conclusion part with all the descriptions and results of this article.

*We apologize for the oversight. We had not properly followed the formatting standard of PLOS One and mistakenly included the conclusions in the "Results and discussion" section. We have added the "Conclusions" section, which includes a comprehensive summary of the topic discussed in the paper, the analyses conducted, and the results obtained.*

7. The author can improve this paper by adding more current and fit references.

*The References section has been completely revised. In the previous version of the manuscript, it was too extensive and scattered. Thanks to the feedback from reviewers that helped to better focus the paper, it is now more fitted and concise.*

**Reviewer #3**: The paper seems very sound in its scientific method. It is also clearly written apart from a few places where the English could be improved (eg p7 line150). My concern has to do with the conceptual framework. In its title the paper claims to engage with "the power of narratives", yet while there are references to scientific literature dealing with "false narratives", nowhere does it offer anything like a systematic theory of narrative. It is hard to know where to begin in offering feedback on how to address this shortcoming, as there are so many different theories of narrative. The authors might consult Tangherlini et al https://doi.org/10.1371/journal.pone.0233879 which offers a good example of how a scientific publication for this journal can manage to combine a full discussion of its quantitative experimental method, while at the same time scientifically laying out a (qualitative) theory narrative, and showing how it applies that theory to its quantitative experiment. This, I think, is the standard to which the journal aspires.

*We agree with the reviewer that the previous version of the paper cited the term 'narrative' without delving into it scientifically. However, this is more of a terminological error than a substantive one, in the sense that the paper does not investigate the concept of narrative; rather, it qualitatively considers the elements: expressed stance and discussed topic.
The suggested reference (*[*https://doi.org/10.1371/journal.pone.0233879*](https://doi.org/10.1371/journal.pone.0233879)*) has been included in the paper, specifically at line 481. This addition aims to offer further insights into conducting a comprehensive analysis of narrative elements.*

*We hope that this revised version clarifies the matter, and we thank the reviewer for highlighting a theoretically important point.*

Finally, a smaller issue has to do with the opening conceptual framework of 'the public sphere', which I do not see as informing the analysis. The primary reference is 60 years old, with the more secondary reference being to a somewhat obscure Marxist figure. There has been so much written on this framework, that I question the need for opening this can of worms at all, when it seems like the paper is more interested in (polarized) political opinion than in the normative public sphere. As I say, the core problem is one of clarifying the framework, with the biggest issue pertaining to having an actual the theory of narrative, and secondarily getting clearer on the whole business of the public sphere and how that highly normative argument informs the argument of the paper as a whole—if the authors want to hold onto the latter, at the very least they'd need to come back to it in the conclusion.

*We sincerely appreciate the reviewer's suggestion. We agree that incorporating the previous conceptual framework of 'the public sphere' does not significantly inform the analysis. As a result, we have thoroughly revised both the Abstract and Introduction, and have omitted the citation from the text.*
