# Supplementary material for: Dynamics and triggers of misinformation on vaccines
Source: PLoS One. 2025 Jan 15;20(1):e0316258. doi: 10.1371/journal.pone.0316258 (PMC11734983; doi:10.1371/journal.pone.0316258)
Supplement: Attachment — Submitted filename: Response to Reviewers.docx [file pone.0316258.s021.docx]

PONE-D-22-33837-R1

**Dynamics and triggers of misinformation on vaccines**

**Response to Editor**

Thank you for submitting your manuscript to PLOS ONE. After careful consideration, we feel that it has merit but does not fully meet PLOS ONE’s publication criteria as it currently stands. Therefore, we invite you to submit a revised version of the manuscript that addresses the points raised during the review process.

The article is considerably implemented compared to its first version. However, a further revision step is necessary in order to further improve the methodological and argumentative aspects (see comments below).

Dear Dr. Andrea Cioffi, PLOS ONE Academic Editor,

Thank you for your feedback and for giving us the opportunity to revise our manuscript. We have carefully addressed all the points raised during the review process, as detailed in the following responses to reviewers.

Kind regards,

Emanuele Brugnoli

**Response to Reviewers**

(Please upload your review as an attachment if it exceeds 20,000 characters)

**Reviewer #1**

1) Why do the authors use 96 of 121 major Italian newspapers, rather than all 121? Can the authors break out this bulleted breakdown in Questionable and Reliable as shown in Table 1? I am most interested to know if all questionable come from online news or not?

To avoid introducing bias in the classification of information sources as reliable or questionable, we relied on the work of third-party organizations (i.e. bufale.net, butac.it, facta.news, newsguardtech.com, and pagellapolitica.it). Therefore, the coverage of the information system analyzed is limited to the sources cataloged by these organizations. We compared the analyzed sample of information sources with known data regarding audience (both digital and non-digital) to support the claim that they have effectively captured almost the entirety of vaccine-related information provided to Italians in recent years.

We also note that our analysis focused on social media. Therefore, among the sources listed by the aforementioned fact-checkers, we only considered those that had at least one active account on one or more of the social media platforms analyzed. Specifically, of the 121 major Italian newspapers identified by GfK Mediamonitor, "only" 96 were present in the lists of the fact-checkers considered and, at the same time, had an active social media account. Note that the 25 missing newspapers are small local papers with low circulation, some of which are in the process of being dismantled.

We added the text *“Except for one TV channel, which primarily operates online as well, all the remaining questionable sources (160) fall under the category of online-only news outlets.”* (page 14, lines 394-395)*.*

2) I am not sure how I am supposed to think of transfer entropy in the pre-pandemic period. I do not know much about Italian vaccine laws, but judging by Figure 1, questionable sources start shooting up on coverage at a very similar time to the reliable sources, peak a still very low number, then peak after the law. For the rest of the time the value is near 0, to the point where inferences are basically built on noise? Meanwhile questionable sources have a valley between their peaks and then drop down before the reliable’ s second peak. I just want to understand if this time period, were all the action is, is driving the entropy results, or are they picking up on the relatively tiny movements over the rest of the timeframe? Either way, it may may sense to isolate that time period more, rather than the full pre-COVID period. I say this assuming that in the pre-COVID time period most of the people consuming anti-vax material were seeking it out with likely high personal demand for the material, making the production less interesting than the in the two times of acute demand during the new law and COVID.

Thank you for your thoughtful comment, which brings up an important point about how to interpret the transfer entropy results during the pre-pandemic period. We appreciate your attention to the possible "noise" in the prevalence of vaccine-related content among reliable sources. In fact, as you pointed out, this aligns with one of the key findings of our work, which we discuss throughout the paper and in particular in the Conclusion: *"Despite the exceptional nature of the Covid-19 event, the spread ease of false claims is only partially attributable to the presence of misinformation sources, and more likely due to the inability of mainstream media to drive the public debate over time on issues that are particularly sensitive and emotional."* This observation is also reflected in the dual-scale representation in Figure 1, which shows the existence of two distinct patterns in the two periods identified in the paper (see the trend of the red curve in the figure, right scale): in the first period, which runs until early 2020, the percentage of content from questionable sources remains flat at around one-third; after the start of the pandemic, this proportion structurally declines to about one-tenth.

Regarding the transfer entropy calculations, it’s important to note that, being computed against a statistical benchmark, the "noise" the reviewer mentions is actually statistically significant in both directions during the pre-pandemic period. Specifically, we observe that the two time-series (reliable and questionable sources) have a significant reciprocal influence, with a similar strength, albeit with a slight advantage for questionable sources. By contrast, during the pandemic, only one direction (R -> Q) remains statistically significant, as shown in Table 2. This change further highlights the shift in dynamics between the two periods and confirms that the reciprocal influence observed pre-pandemic was not simply random noise, but a meaningful interaction between the two sets of sources.

3) E = interactions / (content * followers), and the authors use variations of this to see how content about vaccines and non-vaccines categorization, and/or between publications (or source types) within category do.

3a) I had to spend way too long to figure out exactly what the authors were doing, and how to interpret the plots, and I am still not sure I am reading everything correctly. In the writing the authors are leaning into differential interactions per content & followers, but they have created a pretty convoluted way to do it. I would like the authors to tie the findings in the paper directly to what they are referring to in the figures. I spent too much time searching around, especially as they are referring to specific values that are not directly noted on the charts.

We thank the reviewer for raising this point, which gave us an opportunity to reflect further on how to better describe our findings. The definition of engagement we provide is indeed universal and, in our view, rightly accounts for the audience size of the information source (i.e., followers). In analyzing the impact of misinformation rhetoric on readers (engagement) versus traditional media, we found it valuable to explore both an internal perspective (within questionable or reliable source sets) and an external perspective (between the two source sets) through a comparison metric called “out-engage factor”. The combination of stance conveyed, topic discussed, and types of sources introduces complexity, but we aimed to perform a thorough analysis to support strong claims and assess both the merits of misinformation in terms of engagement and the shortcomings of traditional media.

In response to the reviewer’s feedback, we have revised the figure captions to make them more self-explanatory, and we have enriched the in-text discussion of the figures to ensure that readers do not need to consult the Materials and Methods section beforehand to understand the plots. For this reason, we have also moved the Methods section to follow Results and Discussion. In addition, the axis labels of Figure 4 have been revised to further clarify the log-linear relationship depicted in the plots. We hope these changes improve the readability of the paper and make it easier to follow.

3b) I assume many of the questionable content providers have very low follower counts, which could really impact the key findings?

Thank you for bringing up this important point. Indeed, many questionable content providers have lower follower counts. However, as noted in the manuscript (page 9, lines 233-239), normalization by followers is designed to address this issue in comparing the engaging power among sources with audiences of varying magnitudes. While normalization by followers doesn't affect the internal comparison (as it contributes equally and inversely), it does have a significant impact on the external comparison by either reducing the influence of content from sources with large follower bases or amplifying the contribution from sources with smaller followings. This approach allows us to avoid confusing sheer scale effects with actual user engagement, ensuring that our results reflect genuine interaction with the content rather than just the size of the audience. For these very reasons, social media engagement is, at the industry level, a metric that is always calculated in this way.

For the sake of completeness, we have also included S6 Fig., which illustrates the distribution of followers by category (Questionable vs. Reliable) across Facebook, Instagram, Twitter, and YouTube, as referenced in the main text on page 16, lines 421-423.

3c) I assume higher interactions per content/followers were happening pre-pandemic because the content was more uniquely sensational, then the what was being produced during the pandemic (i.e., it is influenced both the much lower quantity of content and the click-baity nature of it). Could definitely think more about the crowding-out issues that happen during the pandemic.

Thank you for your valuable comment, with which we fully agree. As you rightly pointed out, the Covid-19 pandemic introduced a "crowding-out" effect, where the oversaturation of information likely contributed to a decrease in user engagement with individual pieces of content (both verified and unverified).

Throughout the main text, we recognize the role of the exceptional infodemic that accompanied the Covid-19 outbreak and the immediate vaccine debate in reducing the engagement power of vaccine-related content, as measured in our study by the number of interactions per content/follower. See, for instance, lines 329-332 of page 12: *“While the differences in engagement gained from extreme positions become more pronounced when focusing on the pre-pandemic period, the sudden onset of the Covid-19 pandemic and the subsequent inundation of news about vaccines had a levelling effect, thereby aligning these metrics to comparable values”* and the Conclusion section: *"Our analysis returns a median engagement 6 times higher for misinformation than non-misinformation during the overall period, which rises to 11 when time is limited to before Covid-19 outbreak. [...] The pandemic shock confirms the detrimental effects of the convulsive dynamics of the public agenda on social debates. [...] The Covid-19 pandemic has been an unprecedented event, not just from an epidemiological perspective, but also for the entire information ecosystem. [...] Consequently, misinformation sources have lost their leading role in the public debate on vaccines and have seen a substantial reduction in the engaging power they once exhibited prior to the Covid-19 outbreak."*

I really like the work. The modelling is compelling. And the findings are pretty straight forward descriptive stats, hidden by a needlessly complex framework, that kept making me go back and forth between the methods, writing, and plots.

We thank the reviewer for the kind words of appreciation for our work. We acknowledge that the extensive analyses conducted can sometimes make the paper challenging to follow. Nevertheless, we have followed the reviewer’s suggestions and ensured that the "Materials and methods" section is not strictly necessary for understanding the results discussed. Additionally, we have revised the figure captions to make them more self-explanatory and sufficient for understanding the represented plots. This, in our view, should have made the paper easier to understand, even for readers without expertise in the specific methodologies, and especially without the need to go back and forth between the methods, writing, and plots.

**Reviewer #4**

I find this manuscript very interesting and usefull for the scientific community involved in the study and promotion of the adequate use of vaccines. I am not an exprt in mathematical or deep learning models used in this paper, so I cannot evaluate the methodology used. To my best knowledge, the problem had a correct approach and the results provide important information regarding ways to get involved in proposing convincing counter-narratives to misinformation spread. Very important the results regarding the lack of engagement in the rhetoric of vaccine safety. We all should participate and learn from this study.

We thank the reviewer for the thoughtful and supportive feedback. We are glad to see the importance of our findings recognized, particularly for the scientific community involved in vaccine promotion and combating misinformation.
